# Adenocarcinoma of Urinary Bladder With Distant Metastasis: Huge Fungating Tumor Eroding and Ripping-Off Through Suprapubic Region: A Rare Presentation

**DOI:** 10.7759/cureus.24698

**Published:** 2022-05-03

**Authors:** Shubham Gupta, Jay D Dharamshi

**Affiliations:** 1 General Surgery, Jawaharlal Nehru Medical College, Wardha, IND; 2 Urosurgery, Jawaharlal Nehru Medical College, Wardha, IND

**Keywords:** mucinous subtype, inguinal metastasis, lung metastasis, urinary bladder cancer, adenocarcinoma

## Abstract

Adenocarcinoma of the urinary bladder is an extremely rare entity. It has aggressive behavior and it responds poorly to chemotherapy and radiotherapy. Painless hematuria and passage of clots are mainly common symptoms. Primary bladder adenocarcinoma has a poor prognosis due to its late presentation at an advanced stage. We present here, a rare case report of a 65-year-old male who presented with a huge ulcerative fungating tumor of clinical size 7 x 6 x 5 cm and radiological size 8.6 x 8.1 x 8 cm (exophytic component) and 3.6 x 3.3 x 3 cm (endophytic component) at suprapubic region eroding through pubic bone, rectus muscle and skin with features of lung and inguinal nodes metastasis. Wedge biopsy from fungating growth was suggestive of adenocarcinoma of the urinary bladder of papillary mucin secreting subtype which is also a very rare variant and subtype to get reported. It has a very poor prognosis and only a 6% of survival rate. Keeping all this context, due to poor Eastern Cooperative Oncology Group (ECOG) performance status, huge tumor burden, and advanced presentation of disease, it was suggested by the institutional tumor board, to provide him with the best palliative care management.

## Introduction

Cancer of the bladder, also known as urothelial cancer or urinary bladder cancer, is the 10th most common cancer in the world and its incidence is steadily rising worldwide, especially in developed nations [1,2]. In the lower abdomen, the urinary bladder is a hollow organ whose main function is to store urine coming from the kidney via the ureter until micturition. The urinary bladder is lined by specialized transitional epithelial cells known as uroepithelial cells which accommodate the volume of urine by epithelial flattening under pressure [3]. An estimated 80,500 cases of bladder cancer were diagnosed in the United States (US) in 2019, representing 4.6% of all cancer diagnoses (greater than the global average) which makes bladder cancer the sixth most common cancer diagnosis in the US [4]. Bladder cancer is ranked 10th in incidence in the world. In India, it is ranked 17th in incidence and 19th in mortality [5]. Its incidence rate is found highest in Delhi in both males and females [6]. Adenocarcinoma of the bladder represents 0.5%-2% of all malignant bladder cancers, on the other hand, urothelial and squamous cell carcinoma (SCC) represents 85%-90% and 3%-4% of all bladder carcinomas, respectively [7]. Among subtypes of adenocarcinoma of the bladder, the mucinous subtype is the third most common primary vesical lesion (15%) [8,9]. Patients with exstrophy of the bladder and urachal remnants are at high risk of developing adenocarcinoma of the urinary bladder [10]. There is a hypothesis about the progression pattern from mucinous metaplasia to mucinous adenoma to mucinous adenocarcinoma; however, there is no confirmatory study to date [11]. This cancer has aggressive behavior with late onset of symptoms and delayed diagnosis leading to a worse prognosis. Radical cystectomy with pelvic lymph node dissection remains the main line of curative treatment options. This tumor shows a poor response to chemotherapy and radiotherapy [12,13]. Due to rarity of this tumor and relative lack of clinical reports, we propose this rare case report of adenocarcinoma of urinary bladder of papillary mucin secreting subtype with advanced presentation of huge ulcerative fungating tumor protruding and piercing through all supra pubic structures along with inguinal and lung metastasis.

## Case presentation

A 65-year-old male presented to Urosurgery Outpatient Department (OPD) with complaints of a huge supra pubic lump for four months, which ulcerated into a fungating mass over a period of four weeks; ripping off, protruding, and eroding through all suprapubic structures. He also complained of hematuria for 1.5 months which was insidious in onset and rapidly progressive in nature. He also had associated symptoms of increased frequency of micturition and burning micturition for 10 days. He had a history of nausea and vomiting, no history of fever, pain in the abdomen, breathlessness, jaundice, backache, or any bony ache. He also has no history of any comorbidity (diabetes, hypertension, tuberculosis, sexually transmitted diseases, bronchial asthma). He had a past history of open cystolithotomy done eight years back.

**Figure 1:**
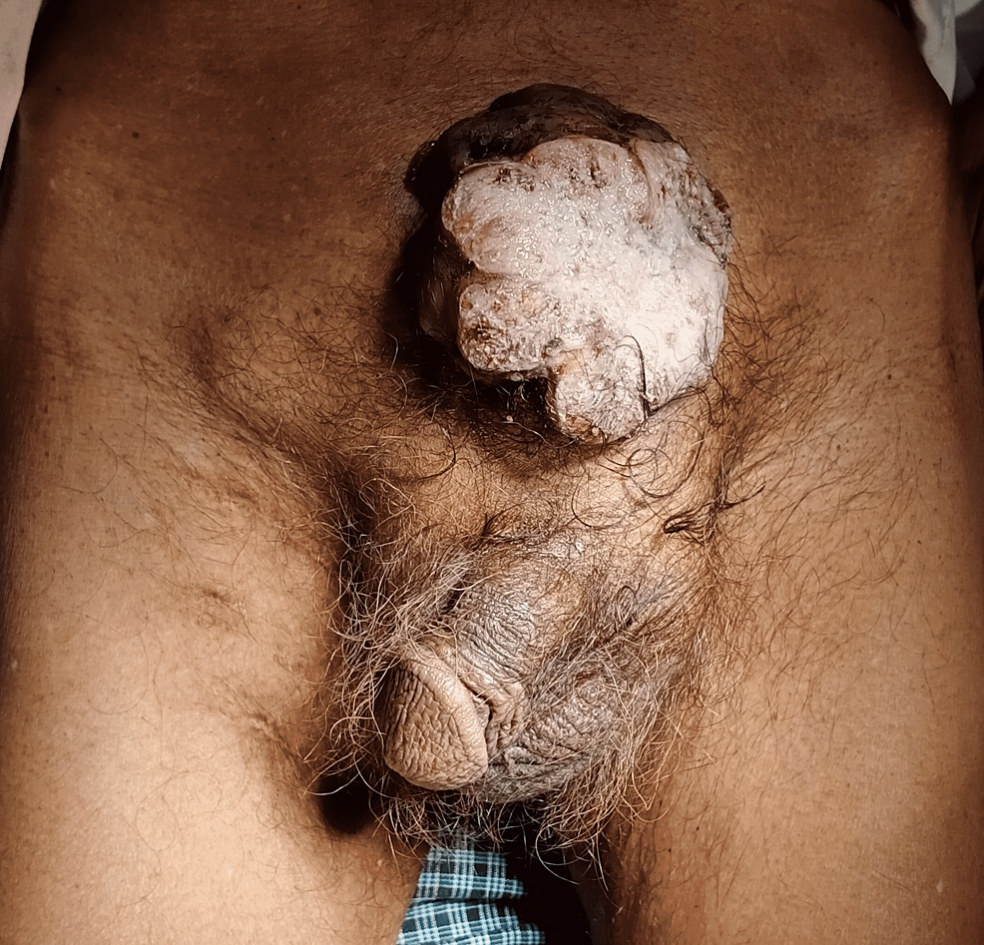
Clinical image of huge ulcerative fungating mass protruding from suprapubic region with right inguinal lymphadenopathy.

On clinical examination, the patient was poorly built with an Eastern Cooperative Oncology Group (ECOG) performance status of 3. He also had significant pallor present but no icterus, cyanosis, or clubbing. Clinically, the patient had a right inguinal lymph node palpable. He was vitally stable. On per abdominal examination, there was a single ulcerative fungating mass of size 7 x 6 x 5 centimeter (cm) in the suprapubic region, fixed to the underlying muscle and bone. It was firm in consistency, friable surface, bleed on touch with right inguinal lymphadenopathy of size 2.5 x 1.5 cm. The mass was non-tender, firm in consistency, and mobile in the vertical direction (Figure 1). On digital rectal examination, he had grade II prostatomegaly with normal rectal mucosa.

**Figure 2:**
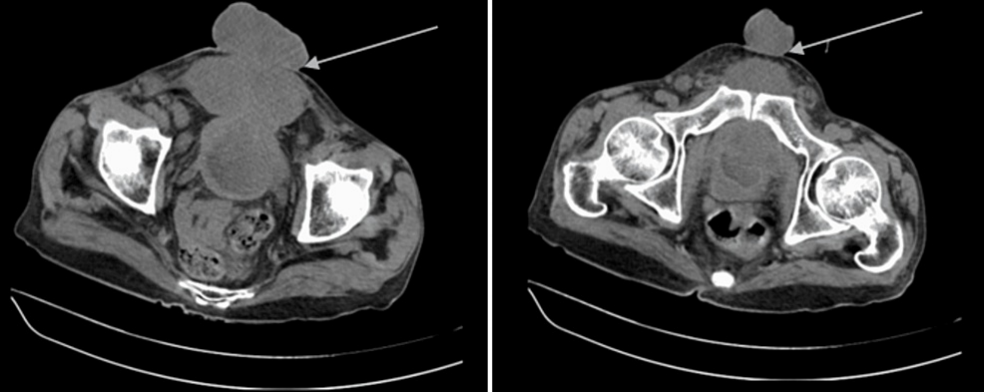
Non-contrast computed tomography scan showing large exophytic and endophytic components of polypoidal lobulated soft tissue mass lesion originating from the wall of urinary bladder and also mild cortical irregularity and erosions in superior pubic ramus and pubic symphysis (left and right).

On routine blood investigation, his kidney function test was urea 76 milligram per deciliter (mg/dL), creatinine 2.6 mg/dL, sodium 141 milli equivalent per liter (mEq/L), and potassium 4.8 mEq/L. Since his creatinine was raised; patient underwent non contrast computed tomography of abdomen which was suggestive of large exophytic polypoidal lobulated soft tissue mass lesion noted in the pelvis with loss of fat planes with antero-superior wall of the bladder, arising from the urinary bladder. The lesion is protruding into the lumen of urinary bladder, suggestive of endophytic component. Bulk of the lesion is present in anterior perivesical space with extension outside the anterior abdominal wall. Posteriorly, the lesion is involving antero-superior wall of urinary bladder and bilateral vesico-ureteric junction with resultant bilateral hydroureteronephrosis. Laterally, the lesion is abutting proximal portion of bilateral spermatic cord. There is mild perivesical and perilesional fat stranding. There is a maintained fat plane between the lesion and seminal vesicles. The exophytic component of lesion measures 8.6 x 8.1 x 8 cm. Endophytic component of the lesion measures 3.6 x 3.3 x 3 cm (Figure 2). Mass effect is noted in the form of displacement of ileal loops superiorly with maintained fat planes with bowel loops. Urinary bladder is partially distended and shows irregular wall thickening up to maximum thickness of 14 millimeter (mm). Suprapubic ramus and pubic symphysis show mild cortical irregularity and erosion by the mass lesion (Figure 2).

**Figure 3:**
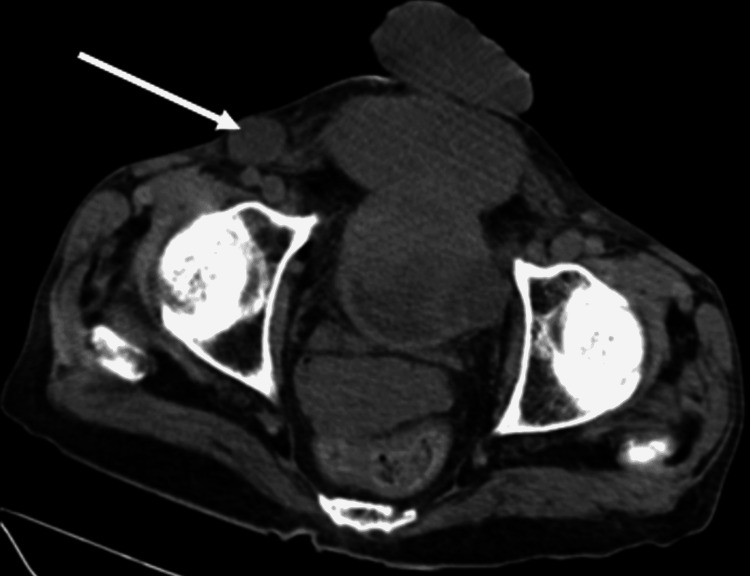
Non-contrast computed tomography scan showing right inguinal lymphadenopathy of largest size measuring 2.7 x 1.6 cm.

Multiple enlarged lymph nodes in right inguinal region with loss of fatty hilum, largest measuring 2.7 x 1.6 cm (Figure 3).

**Figure 4:**
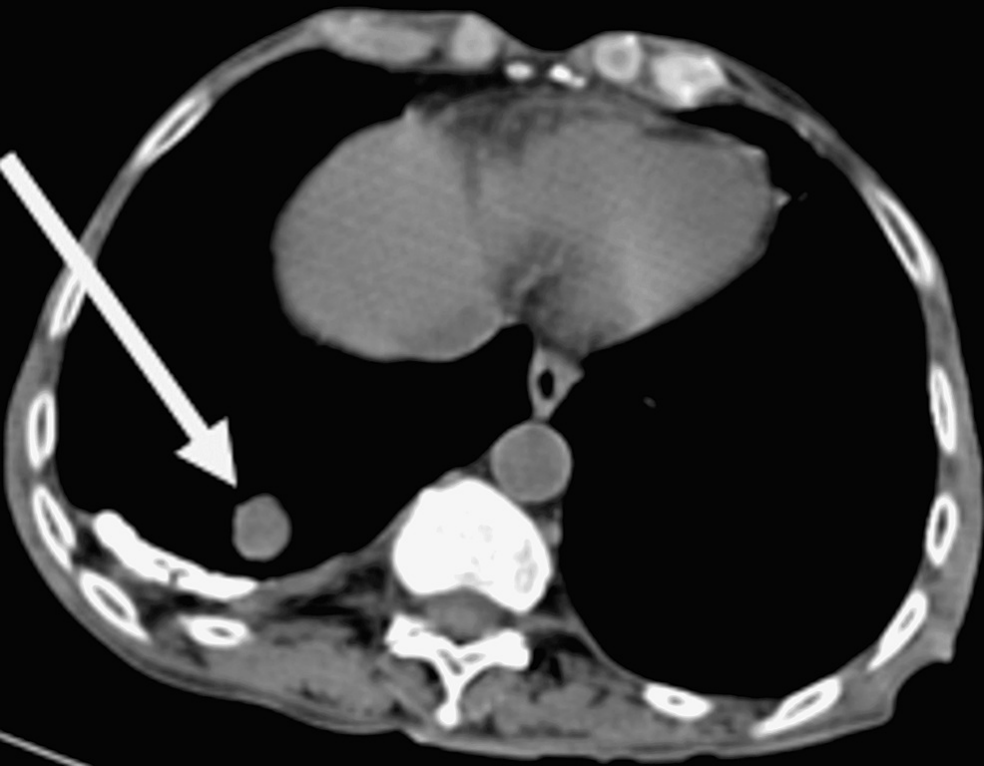
Non-contrast computed tomography scan showing lung metastasis with largest size of metastatic nodule measuring 17 x 18 mm in poster basal segment of right lower lobe.

Multiple variable-sized soft tissue lesions with spiculated margins in bilateral lung parenchyma, largest measuring 17 x 18 mm in posterobasal segment of right lower lobe suggestive of metastasis (Figure 4).

**Figure 5:**
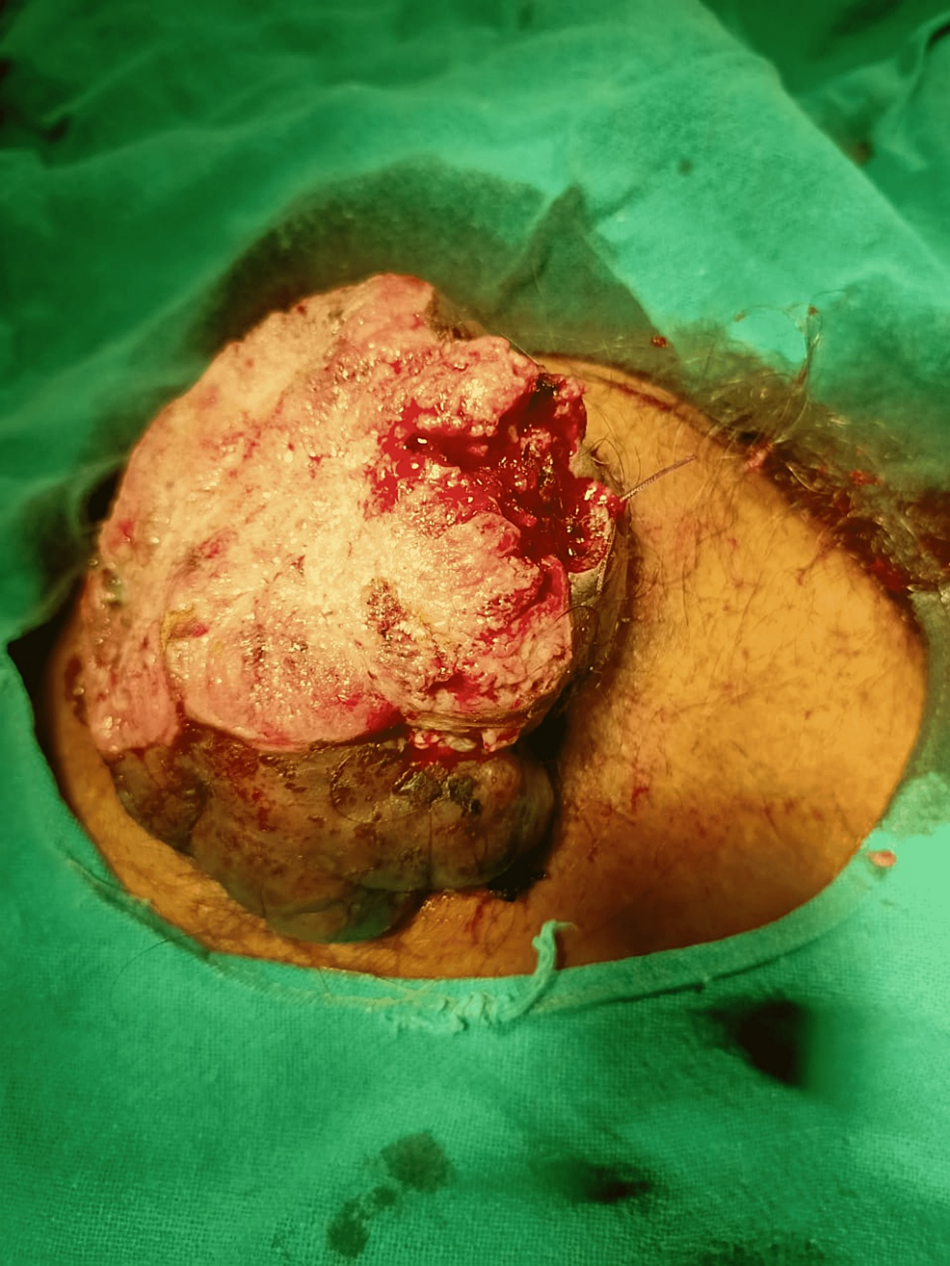
Clinical image of fungating tumor after taking wedge biopsy from it.

**Figure 6:**
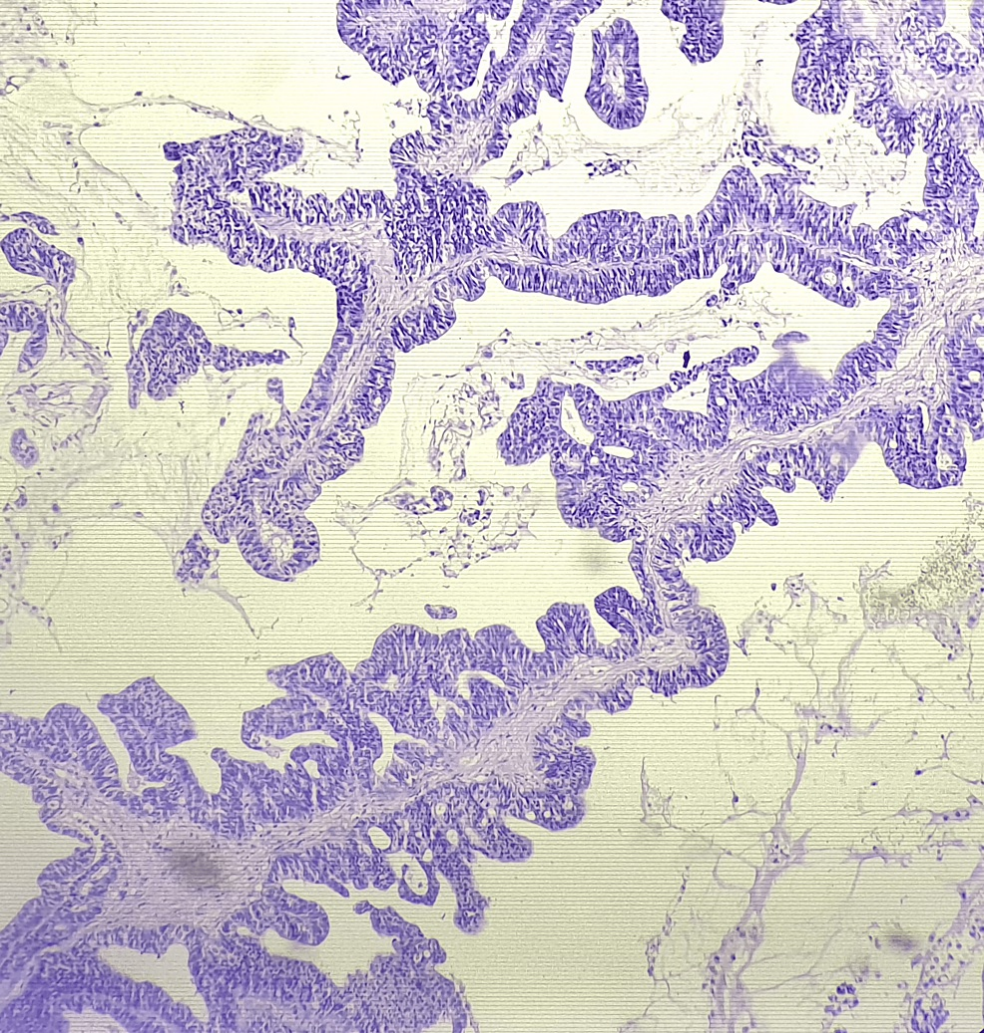
Histopathological image of the edge biopsy of fungating ulcer from hypogastric region showing well-differentiated adenocarcinoma of bladder (papillary mucin type) under 10x view.

**Figure 7:**
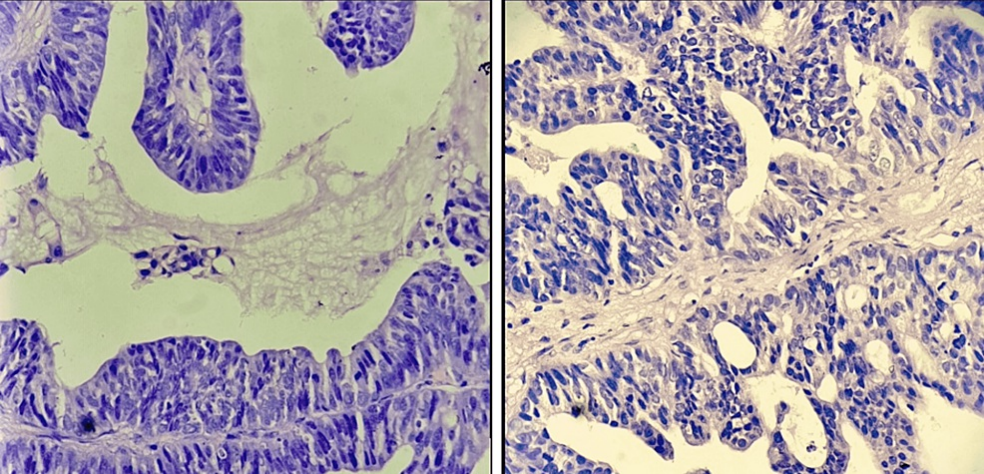
Histopathological images (left and right) of the edge biopsy of fungating ulcer from hypogastric region showing well differentiated adenocarcinoma of bladder (papillary mucin type) under 40x view.

Wedge biopsy (Figure 5) from that fungating ulcer was suggestive of well differentiated adenocarcinoma of papillary mucin secreting subtype (Figures 6, 7).

Institutional Tumor Board Discussion (TBD) was done and palliative treatment was advised, considering ECOG performance status of patient to be 3, deranged kidney function and huge tumor burden with advanced presentation of the adenocarcinoma of bladder with multiple right inguinal lymphadenopathy and distant lung metastasis. 

## Discussion

Adenocarcinoma is a rare histological variant that accounts for 0.5%-2% of urinary bladder carcinoma in the United States [14]. Patients with adenocarcinoma are usually found between the sixth and seventh decade of life with more male predominance. Such patients mainly present with hematuria as the most common symptom, but some of the patients also present with bladder irritation as a symptom, and rarely some present with mucusuria as well [15]. At the time of presentation, approximately one-third of the patients present with lymph node metastasis [16,17]. In this case also, the patient was a 65-year-old male with hematuria as an associated complaint and right inguinal lymphadenopathy.

The pathogenesis of bladder adenocarcinoma in although not yet understood but mostly, almost 90% of bladder tumor patients with exstrophy growth of the bladder are adenocarcinoma [14]. According to the literature and previously reported cases of bladder cancer; palpable bladder, suprapubic pain, left renal or left flank pain, macroscopic hematuria and dysuria have been noted as presenting complaints of the patients respectively [18,19]. While there has been an enormous scarcity or almost negligible evidence of bladder cancers with “huge ulcerative fungating bladder mass of clinical size 7 x 6 x 5 cm (Figure 1) and radiological size 8.6 x 8.1 x 8 cm (exophytic component) and 3.6 x 3.3 x 3 cm (endophytic component) respectively, eroding and ripping off all suprapubic structures” (Figure 2) reported in literature till date. We report this case of bladder cancer with an extremely uncommon presentation with huge fungating bladder mass eroding, projecting out, and piercing through all suprapubic structures (Figure 1).

The association between urinary bladder stones with SCC of the bladder is well established in the literature and vesical stones have been considered a risk factor for SCC of the bladder [20-22]. Alsheikh et al. [23] have also reported an alliance or interrelationship of bladder stone with an aggressive plasmacytoid variant of urothelial bladder cancer which proved to be a quite rare variant among all subtypes of bladder cancers. Our patient had a history of open cystolithotomy done eight years back in 2014 for a urinary bladder stone. Later then he presented with a huge supra pubic lump for four months which got ulcerated into fungating mass, eroding and ripping off through all suprapubic structures for four weeks. Wedge biopsy (Figure 5) was done which was suggestive of well-differentiated adenocarcinoma of the urinary bladder which is of papillary mucin secreting subtype. To our knowledge, this would be the first rarest case to be reported in the literature exhibiting an association of bladder stone with adenocarcinoma of the urinary bladder.

On histological examination, adenocarcinoma of the bladder may show various growth patterns like (i) enteric (colonic or intestinal) (5%); (ii) mucinous/colloid (15%); third most common (iii) signet ring cell (22.5%); second most common; (iv) not otherwise specified (NOS) (52.5%); most common; and (v) mixed pattern [9,15]. In our patient, papillary mucin secreting type of growth pattern (Figures 6, 7) was noted in histopathology, which stands to be the third most common variant among all types of growth patterns observed in adenocarcinoma of the bladder.

Shinagare et al. [24] have demonstrated a pattern of metastasis in the case of bladder cancer; lymph nodes, bones, lungs, liver, and peritoneum are the most common sites for metastasis from carcinoma of the urinary bladder. Ravikumar et al. [25] reported a case of adenocarcinoma of urinary bladder with metastatic malignant pleural effusion. In our patient, computed tomography scan of chest (Figure 4) similarly revealed lung metastasis with largest size of metastatic nodule measuring 17 x 18 mm in posterobasal segment of right lower lobe.

Metastasis to inguinal lymph nodes in cases of bladder cancer is very rare scenario. Only few cases (4-5) of such inguinal metastasis have been reported in the literature and that to after radical cystectomy in cases of transitional or urothelial carcinoma of bladder [26,27]. Patient in our case of adenocarcinoma demonstrated clinically palpable right inguinal lymphadenopathy (2.5 x 1.5 cm) (Figure 1) as well as radiologically revealing multiple enlarged right inguinal lymph nodes with loss of fatty hilum, largest measuring 2.7 x 1.6 cm, prior to any interventional therapy (Figure 3).

Most of the primary bladder adenocarcinoma patients have muscle invasion and are treated with radical cystectomy and pelvic lymph node dissection [28,29]. Several retrospective studies have shown that adenocarcinoma of bladder with very poor clinical outcome as compared to urothelial carcinoma of bladder [17,30,31]. In case of our patient, institutional TBD was done and advised palliative care management, considering ECOG performance status of patient to be 3, deranged kidney function and huge tumor burden with advanced presentation of the adenocarcinoma of urinary bladder of mucinous subtype with distant lung and inguinal metastasis.

The prognosis and outcome of adenocarcinoma of bladder is very poor due to late diagnosis of its advanced stage [10]. The survival rate of bladder cancers mentioned in the literature in cases of distant metastasis is only 6% [32]. Our patient who was on palliation therapy for his disease, survived only for two months.

## Conclusions

Adenocarcinoma of the urinary bladder is an aggressive type of bladder tumor. In the literature, only a few cases exhibiting association of adenocarcinoma bladder with bladder stones have been reported. Our patient’s unusual presentation with a history of bladder stone removal in past and adenocarcinoma of the bladder highlights the possible interrelation between these two conditions.

Presentation of bladder tumors (of any type) in the form of such a huge fungating mass eroding, splitting up, and protruding through suprapubic structures has never been reported yet. This might be the first such case to get reported with such a rare clinical presentation. While dealing with the cases of bladder cancers, awareness regarding this variant, adenocarcinoma with a mucinous subtype of bladder cancer must be kept into consideration.

The treatment guidelines or protocol for managing adenocarcinoma of the urinary bladder varies according to the muscle invasion and staging of the tumor. To date, limited available data suggests adenocarcinoma is an aggressive type of bladder tumor with poor clinical outcomes and prognosis.
